# Nutrition as a Modifiable Factor in Optimizing Respiratory Health: Evidence from Pulmonary Function Tests

**DOI:** 10.3390/children13040543

**Published:** 2026-04-14

**Authors:** Paraschiva Chereches-Panta, Daniela Pop, Claudia-Felicia Pop, Marcela Daniela Ionescu, Alina Petronela Bouari-Coblișan, Valentina Sas

**Affiliations:** 1 Department 8, Mother and Child, Third Pediatric Discipline, Faculty of Medicine, “Iuliu Hatieganu” University of Medicine and Pharmacy, 400124 Cluj-Napoca, Romania; pusachereches@umfcluj.ro (P.C.-P.); sas.valentina@umfcluj.ro (V.S.); 2 Third Pediatric Clinic, Clinical Hospital for Pediatric Emergencies, 400315 Cluj-Napoca, Romania; petronela.coblisan@umfcluj.ro; 3 General Nursing Discipline, Department 1, Faculty of Nursing and Health Sciences, “Iuliu Hatieganu” University of Medicine and Pharmacy, 400124 Cluj-Napoca, Romania; felicia.pop@umfcluj.ro; 4 Department of Pediatrics, “Carol Davila” University of Medicine and Pharmacy, 020021 Bucharest, Romania; daniela.ionescu@umfcd.ro; 5 Emergency Clinical Hospital for Children “M.S. Curie”, 041451 Bucharest, Romania

**Keywords:** pulmonary function, FEV1, micronutrients, Mediterranean diet, Western diet, vitamin D deficiency, iron deficiency, asthma, children

## Abstract

**Highlights:**

**What are the main findings?**

- A knowledge gap exists regarding the real-life dietary habits of asthma patients.
- Current concerns add to the multifaceted picture and multidimensional implications of asthma patient care.

**What is the implication of the main finding?**

- Adherence to the Mediterranean diet correlates with higher FEV1 and FVC values, while an increase in the dietary inflammatory score decreases FEV1.
- Maternal diet may have effects on lung health, including respiratory function in children. Vitamin A, vitamin E, zinc, and selenium are correlated with better FEV1 and FVC.

**Abstract:**

**Introduction**: Pediatric asthma is the inflammatory condition with the highest burden of chronic disease in children. Awareness of the undesirable effects of modern lifestyles, including sedentary behavior and eating habits associated with Western diets, has led to novel approaches in clinical practice. Current concerns focus on the possibility of non-pharmacological intervention to achieve better disease control and normal lung function in these children. **Method**: In this narrative review, we analyzed current information on the influence of dietary patterns on lung function. The aim was to clarify the extent to which current knowledge provides arguments for applying certain dietary measures to asthma patients in order to optimize lung function. We conducted research in the literature to evaluate the impact of Western diet, Mediterranean diet, and micronutrients status on lung function. We also focused on how maternal diet during pregnancy can influence lung function in offspring. **Results**: We found a positive impact on lung function in children who adhere to the Mediterranean diet, in contrast to the Western diet which is related to low asthma control. Deficits of micronutrients like selenium, zinc, iron, and vitamin D are linked to impaired lung function. Maternal intake of fiber, vitamin A, vitamin E, zinc, and selenium during pregnancy is correlated with better FEV1 and FVC. However, current information on this topic is controversial, and there is no clear data on intervention measures in clinical practice. **Conclusions**: Evaluation and clear recommendations of diet could contribute to a better management of children with asthma.

## 1. Introduction

The pathogenesis of asthma is complex and involves a combination of genetic and environmental factors, including air pollution and lifestyle factors. Recent studies are placing increasing importance on lifestyle and dietary changes as relevant aspects that may have an impact on asthma prevalence and disease control [1,2]. Nutritional factors may be involved in triggering asthma and other allergic diseases, promoting inflammatory status, affecting lung function, and preventing disease control, with a poor quality of life.

In children aged between 5 and 14, asthma is among the top 10 most common conditions [3]. Most cases are diagnosed during early childhood [4]. Approximately 300 million people worldwide suffer from asthma [5]. In Romania, the last epidemiological data showed a prevalence of asthma of 7% in children [6] with an increasing trend as compared with the first International Study of Asthma and Allergies in Childhood [7]. The Global Asthma Network study analyzed both the prevalence trend of asthma and the severity of the disease [8]. 

Studies have linked the increase in asthma prevalence to the widespread adoption of the Western diet [9,10]. Replacing vegetables, fruits, and grains with a diet predominantly consisting of meat and animal products, milk, and saturated fats may have an effect on the immunopathogenesis and pathophysiology of asthma [9].

A knowledge gap exists regarding the dietary habits of asthma patients in real life. Current concerns add to the multifaceted picture and multidimensional implications of asthma patient care.

We conducted a literature review using the keywords: “micronutrients”, “vitamin D”, “Mediterranean diet”, “Western diet”, “pulmonary function”, “children”, and “asthma”. We searched Scopus, PubMed, Embase, Medline, Web of Science Scholar, and Google through September 2025. We included only English-language articles, randomized clinical trials, longitudinal studies, cross-sectional studies, and meta-analyses relevant to the topics analyzed. We excluded non-English articles, abstracts only, and case reports. The main objective was to determine whether there is a relationship between a child with asthma and their diet, including micronutrient intake and lung function. Identifying a causal relationship would provide the rationale for applying specific dietary measures to optimize lung function parameters and control disease. A secondary objective was to examine the potential role of the mother’s diet during pregnancy in the development of asthma and its possible influence on the offspring’s lung function.

## 2. Pulmonary Function Tests

Chronic inflammation of the airways and bronchial hyperresponsiveness (BHR) are the hallmarks of asthma. The diagnosis is supported by a history of recurrent suggestive symptoms correlated with evidence of BHR [11]. This can be demonstrated by testing pulmonary function. Spirometry, plethysmography, multiple breath washout (MBW), respiratory oscillometry, and peak expiratory flow (PEF) variation measured by ambulatory peak flowmetry are standardized techniques.

Among the available methods, spirometry is the most used in practice. It is reliable, non-invasive, relatively simple, and requiring patient cooperation to perform a measurement that can be correctly interpreted [12]. Spirometry testing and interpretation is regulated by international guidelines [12,13,14]. The degree of airflow obstruction is classified based on forced expiratory volume in one second (FEV1) and forced vital capacity (FVC). To assess small airway function, we can use maximal mid expiratory flow (MMEF), which is the average flow between 25% and 75% of FVC [15]. Values of FEV1 below a predicted 80%, whether or not correlated with an FEV1/FVC ratio below 70% and/or MMEF <65% of the predicted value, indicate bronchial obstruction [16]. The correlation between FEV1 and MMEF and the level of asthma control or the risk of exacerbation is a topic of ongoing interest [15,17]. In patients with uncontrolled asthma, FEV1 may normalize after treatment, while MMEF improves inconsistently [18,19]. Distal airway obstruction can be better assessed using oscillometry and/or MBW [18,20,21].

The monitoring plan for asthma patients includes symptom scores and self-assessment questionnaires on control and quality of life, lung function including post-bronchodilator FEV1 tests, and biomarkers of Th2-type inflammation such as fractional exhaled nitric oxide (FeNO) and eosinophilia [22].

## 3. Decade of Action on Nutrition 

The United Nations General Assembly declared 2016–2025 the Decade of Action on Nutrition, later extending it to 2030 [23,24]. This recognizes nutrition’s impact on health and the need for awareness among governments, civil society, academia, business, and other stakeholders. It was even suggested that the epidemic of asthma in developed countries might be linked to dietary patterns [25]. Evidence linking diet and pulmonary function may inform future guidelines.

Diet has been linked both positively and negatively in relation to asthma and pulmonary function. For example, children with food sensitization in the first two years of life are at higher risk of recurrent wheezing or asthma [26]. Furthermore, a large population-based cohort, longitudinal study showed that children with two or more food allergies were almost three times more likely to develop asthma or recurrent wheezing compared to children with no food allergies [27]. Dietary micronutrients, macronutrients, or dietary patterns may play a role in altering pulmonary function parameters and in the prevention or development of asthma or recurrent wheezing [28].

The epidemics of asthma and obesity show a parallel increase [29]. Unhealthy dietary patterns have been associated with both asthma and obesity [29]. Consequently, improving eating habits and promoting physical activity increases benefits for both conditions [30].

Current data support the idea that dietary changes can have an impact on the immune response and may correlate with the progression of chronic disease [2]. The role of nutrients in immunity is well known. Micronutrients, vitamins A, B6, B12, C, and D, along with minerals and antioxidants, influence innate immunity and inflammatory processes [31]. Nutritional deficiency of these components may be correlated with the onset of asthma [2].

## 4. Nutritional Habits and Pulmonary Function in Children

### 4.1. Mediterranean Diet

The Mediterranean diet is based on vegetables, fruits, nuts, legumes, grains, fish, and olive oil, and low intakes of meat, including poultry, dairy products, and saturated fats. It is rich in vitamin E and omega-3 polyunsaturated fatty acids (PUFA), with a low intake of omega-6 fats. The benefits of this diet may be explained by the modulation of the pulmonary response to oxidative stress, the decrease in the level of pro-inflammatory mediators such as tumor necrosis factor alpha [32,33], and antioxidant capacity [34], by protecting against oxidant-induced lipoperoxidation of PUFA in cell membranes [33,35].

- Mediterranean diet and pulmonary function

Increased intake of fruits and vegetables may reduce bronchial inflammation due to the effect of antioxidants such as vitamin C, flavonoids, and carotenoids, as well as other biologically active phytochemicals [36]. The reduction in inflammation could be assessed by measuring IL-8 in nasal lavage. It was correlated with better FEV1 and FVC values, while IL8 concentration was lower as fruit and vegetable consumption scores were higher [33]. In 2009, Romieu I et al. [33] published the results of a prospective cohort study conducted on a group of 158 children with asthma aged between 6 and 14 years, in which the role of the Mediterranean diet on lung function was analyzed. The results showed that adherence to the Mediterranean diet led to higher FEV1 and FVC values, and that this functional improvement was proportional to the Mediterranean diet index and the fruit and vegetable index. Analysis of bronchial inflammation markers (FeNO, exhaled breath pH, and IL-8 levels in nasal lavage) showed no effect of the diet [33]. When the harmful effect of ozone exposure was analyzed, the Mediterranean diet consumption proved to have a protective effect on FVC and IL-8 levels. A case–control study in children in Peru did not demonstrate an association between the Mediterranean Diet Score (MDS) and lung function, specifically FEV1 [37]. A recent cross-sectional study on the effect of omega-3 PUFA consumption showed that FEV1, FVC, PEF, and FEV25–75 values were higher in those with higher omega-3 PUFA intake [38]. The study included 9378 adults who were interviewed about their consumption of omega-3 PUFA in the previous 24 h. However, after excluding confounding factors such as smoking, omega-3 PUFA consumption was not correlated with lung function [38].

The relevant studies on the correlation between pulmonary function and Mediteranean diet are summarized in Table 1.

### 4.2. Western Diet

The Western diet has been linked to an increased risk of diabetes, cancer, cardiovascular disease, dysbiosis, and alterations in gene expression and epigenetics [30]. It is high in saturated fat, processed food (meat), and refined carbohydrates. The amount of consumed fruits, vegetables, and seafood is low. This leads to an inadequate intake of antioxidants, vitamins, minerals, polyphenols, flavonoids, carotenoids, long-chain omega-3 PUFA, docosahexaenoic acid, eicosapentanoic acid, and docosapentaenoic acid and high intake of saturated fat and omega-6 PUFA, sugar, and sodium [40]. A Western-type dietary pattern is associated with obesity and overweight [41]. Asthma associated with overweight and obesity is a specific phenotype of asthma, often difficult to treat [42]. 

Ready-to-eat cereals for children have high contents of sugar, fat, and sodium [43]. These characteristics are presumed to promote a pro-inflammatory environment. Still, a link between diet-triggered systemic inflammation and airway inflammation needs to be established [40]. In adults with asthma, a higher dietary inflammatory index (DII) was observed compared with healthy controls [44]. A worsening of asthma in adult patients was also associated with high consumption of cured meat [45]. The proposed mechanism is lung damage from nitrosative and oxidative stress induced by nitrate in cured meat [46].

- Western diet and asthma

Most studies in children report positive or negative correlations between a Western dietary pattern and asthma diagnosis or impact on symptom frequency and severity, rather than on pulmonary function measurements. Emrani et al. found, in a group of 7667 children, that 324 children with asthma (mean age ± SD = 11.7 ± 2.94 years) were more likely to have wheezing among boys who adhered to a Western diet [47]. In adolescents aged 13 to 17 years from Taiwan, Huang et al. evaluated the association between dietary factors and asthma and allergic rhinitis [48]. A diet rich in protein and fat from animal sources, mainly meat and liver, and deep-fried foods was associated with a higher prevalence of asthma [48]. 

Frequent wheezing was significantly associated with the Western diet at ages 3 and 4 years [49]. Patel et al. found a significant association between asthma and Western dietary patterns in children aged 8 years, and this association increased the risk of asthma at 11 years [50]. A high DII was positively correlated with elevated plasma IL-6 levels, suggesting systemic inflammation. For every 1-unit increase in dietary inflammatory score, there was a 3.44-fold decrease in FEV1 [44]. A positive association has been described between dietary advanced glycation end products (meat and saturated fats), characteristics of a Western diet, and an increased likelihood of wheezing [51].

A study on adolescents from Brazil showed a positive association of asthma and wheezing and consumption of ultra-processed products in a dose–response manner [52]. Ultra-processed foods may contribute to low-grade inflammation also through non-nutritional components and additives [41,53].

- Western diet and pulmonary function

Obese children have been shown to have a low FEV1/FVC ratio [54], as well as lower functional residual capacity, residual volume, and expiratory reserve volume [55,56]. In children aged 13 years, a higher subcutaneous and visceral mass was associated with lower spirometry parameters (FEV1, FEV1/FVC, and FEF75) [57].

A high DII was positively correlated with elevated plasma IL-6 levels, suggesting systemic inflammation. For every 1-unit increase in dietary inflammatory score, there was a 3.44-fold decrease in FEV1 [44]. Han et al. compared 351 children with asthma with 327 without asthma regarding dietary patterns, lung function, and questionnaire responses. A diet rich in vegetables and grains and low in sweets and dairy products was associated with a higher lung function [25]. Table 2 summarizes studies that assessed correlations between the Western diet and pulmonary function tests.

It was presumed that childhood diet patterns could influence long-term respiratory function [58]. The patients’ dietary patterns were followed prospectively from birth, using questionnaires and objective measures. The study correlated three dietary patterns in childhood (“health-conscious”, “traditional”, and “processed”) with lung function in adolescence. FEV1 and FVC were linearly positively associated with a ”health-conscious” dietary pattern and non-linearly negatively correlated with the ”processed” diet. There was no evidence of an association between the three diets and incident asthma. This study concluded that a diet high in processed foods was associated with lower lung function, while a healthy diet was associated with higher lung function [58].

Rodriguez et al. included 660 children aged 7–12 years in a cross-sectional study [59]. Data on food and beverage consumption were collected using a 24 h recall questionnaire, and the quality and sustainability of the diet was assessed using the Planetary Health Diet Index. Airway inflammation was assessed by FeNO. The authors report a protective effect of a sustainable, healthy diet on airway inflammation in children who are not obese or overweight, but not on asthma.

A study that included 3204 participants (average age 11 years) assessed dietary patterns using a validated questionnaire [60]. For pulmonary function evaluation, the primary measure was the FEV1/FVC ratio. Plant food patterns, fruits, pickled foods, and dietary fiber were found to have a protective effect against expiratory airflow limitation, but only the plant food pattern had a statistically significant association [60].

Western diets are regularly linked to small but clear drops in children’s lung function, especially FEV_1_. Studies over time show these diets may slow lung growth, while other studies suggest a higher chance of airflow issues. Even though the changes are minor, their steady, ongoing presence suggests that Western diets can harm the airways early, potentially leading to long-term breathing problems.

### 4.3. Cow Milk and Dairy Products 

In a large epidemiologic study of school-aged children, Loss et al. [61] found that farm milk consumption was associated with a reduced risk of asthma and allergies, independent of concomitant farm exposure [61]. They found no relation between fat content and viable bacterial count of the milk and asthma or allergies. Higher levels of α-lactalbumin, β-lactoglobulin, and bovine albumin in the milk were inversely associated with asthma, but not with atopy [61]. Raw milk consumption was also found to have protective effects against respiratory infections [62].

A Polish study found that early exposure to unpasteurized milk may protect against allergies and asthma in both children and adults [63]. Other studies also demonstrated raw milk’s protective effect on asthma and atopy [64,65]. Supporting these findings, Wijga et al. found that consuming milk, milk products, and milk fat correlated with lower rates of wheezing and asthma in preschool children [66]. In children aged 6 years, the risk of asthma was reduced among those consuming unprocessed milk, which has a higher content of anti-inflammatory precursor mediators, ω-3 PUFA [67]. In 2020, Brick et al. published a meta-analysis of all studies assessing the protective effect of raw milk against asthma and allergies, as well as the molecules involved in this effect [68]. A recent meta-analysis by Song et al. included 13 studies of children with asthma that assessed the effects of milk and dairy products on asthma. Although the authors found no correlation between reduced asthma risk and dairy product intake, they observed in the non-Asian population a significant association between reduced asthma risk and higher milk and dairy product consumption [69]. Conversely, the risk of asthma or recurrent wheezing decreased in children who avoided cow milk formula in the first 3 years of life when compared with those consuming cow milk formula from the first day of life [70]. Heat-treated milk products and infant formula consumption have been linked to the development of asthma by age 5 [71].

A recent study shows that for children with cow-milk protein allergy, extensively hydrolyzed formulas with a probiotic (*Lactobacillus rhamnosus G*) have a therapeutic effect and, long term, protect against atopic manifestations, including asthma [72].

Common food allergens that trigger respiratory reactions are egg, milk, peanuts, fish, soy, and shellfish [73]. Milk contains numerous components such as long-chain PUFA [67,74], prebiotic oligosaccharides [75], β-glucan [76], lactoferrin, immunoglobulins, whey proteins [77], and cytokines (transforming growth factor beta, TGF-β) [64]. These components have been shown to have a protective effect against respiratory infections and/or allergies [78], by reducing eosinophilic inflammation and type 2 cytokine production. Children aged 1 to 4 years who consumed a cow milk-based beverage containing docosahexaenoic acid, a prebiotic, and β-glucan for 28 weeks had fewer episodes of allergic skin and respiratory manifestations [78]. TGF-β promotes epithelial barrier functioning, and bovine immunoglobulins bind virus and bacterial pathogens, stimulating phagocytosis [65]. TGF-β1 and TGF-β2 are similar in cow and human milk, but TGF-β1 levels decline significantly in processed milk [65]. Some other components, like lactoferrin and immunoglobulins are reduced by the processing of milk [65]. Epigenetic mechanisms might also explain the association between raw milk consumption and allergies and asthma [79].

- Effect of cow milk and dairy products on pulmonary function

Considering the well-known myth that milk products increase mucus production in the respiratory airways, Haas et al. designed a study to objectively assess airway obstruction by measuring airway resistance, which would occur in this circumstance. They found no significant change in FEV1 or forced expiratory flow at 50% of FVC (FEF50) after milk ingestion, neither in asthmatic patients nor in healthy controls [80].

In a study by Nguyen et al., adult atopic patients with mild asthma who ingested cow’s milk did not report acute or delayed symptoms. Patients had no deterioration of the measured pulmonary function tests, FEV1 and FEV1/FVC [81]. Conversely, in a study published by Pelikan et al. [82] in adult asthmatic patients, a food challenge with natural foods and a double-blinded, placebo-controlled study triggered a late asthmatic response to milk, as demonstrated by objective parameters such as spirometry. 

James et al. report that 320 highly atopic patients aged 6 to 30 years were also assessed for the degree of pulmonary dysfunction caused by exposure to food allergens, including cow’s milk; 15% of patients developed respiratory symptoms, but only 6 of them had a >20% decrease in FEV [83].

Koren et al. conducted a prospective, randomized, placebo-controlled, double-blind study to assess the effects on respiratory symptoms, pulmonary function, and oxygenation of a single exposure to cow’s milk in children aged 6 to 18 years, with and without asthma [84]. The study also aimed to dismantle the common belief that cow milk consumption causes respiratory symptoms. As a result of this belief, many parents of children with asthma restrict dairy products from their diet. FEV1 and FeNO were measured before and at different intervals of time within 120 min after the ingestion of milk. The study found that exposure to cow’s milk is not associated with respiratory symptoms, bronchial constriction, or airway inflammation, neither in asthmatic nor in non-asthmatic children [84].

Cow’s milk consumption is generally safe and not associated with worsened respiratory health in the general population; it may even confer protective immune effects under certain conditions (particularly with minimally processed milk in early life). However, these benefits must be weighed against the safety concerns of raw milk and are not applicable to individuals with cow’s milk allergy, in whom adverse respiratory reactions are well established.

### 4.4. Effect of Dietary Egg

Eggs are an accessible source of nutrients and bioactive substances (choline, lutein, zeaxanthin) with multiple biological roles. Vitamins B, D, and A, along with essential fatty acids, antioxidants, and minerals (phosphorus, selenium, iron, iodine, zinc), contribute to the qualities of this food. The increased bioavailability of nutrients in eggs could have beneficial health effects. A recent umbrella review was unable to establish an association between egg consumption and mortality from respiratory diseases [85]. In patients with chronic obstructive pulmonary disease, increased egg consumption has been shown to correlate with a lower dyspnea score [86]. In these patients, a ketogenic diet in which fruits, vegetables, and grains are replaced by foods rich in protein and fat, including increased egg intake, appears to have a beneficial effect on lung function [87]. 

The effect of egg consumption on lung function in children has been reported in only one study comparing 13 children with asthma to 9 children in the control group. The intervention was to restrict both eggs and milk. Sensitization was monitored by measuring specific IgE and PEF. A significant increase in PEF was observed after dietary restriction [88].

### 4.5. Fruit, Vegetables, and Fiber

Polyphenols are chemical compounds found in plants, classified according to their structure into four main types: flavonoids, phenolic acids, lignans, and stilbenes. The main sources of polyphenols in the human diet include fruits, vegetables, seeds, grains, nuts, olives, tea, coffee, and chocolate [89]. The flavonoids that have been extensively researched are quercetin, kaempferol, and myricetin.

The precise molecular and cellular mechanisms by which polyphenols act are complex but not fully understood. They can modulate the local and systemic immune response and influence the composition of intestinal microbiota. Their binding to food allergens reduces the allergen’s ability to bind to IgE. They can intervene in the presentation of the allergen by the dendritic cell, thus reducing the differentiation of CD4+ cells and the occurrence of proallergic cytokines (IL-4, IL-5, IL-9, IL-13) [89].

A series of polyphenols inhibit the production of thymic stromal lymphopoietin (TSLP) and IL-33. Quercetin has been shown to reduce serum levels of IL-25, IL-33, and TSLP and the expression of these cytokines in lung tissue [90]. In experimental models of allergic rhinitis and asthma, a decrease in the concentration of TSLP and IL-33 in nasal and bronchoalveolar lavage fluids has been demonstrated under the action of polyphenols from the Fallopia japonica plant [91].

Some polyphenols decrease antigen-specific IgE production, and can bind to the α chain of FcεRI, preventing IgE binding and consequently reducing mast cell sensitization and degranulation, or can even reduce mast cell degranulation by reducing the expression of calcium channel proteins and blocking calcium influx into the cell [89]. Curcumin decreases the secretion of IL-4, IL-5, and IL-13, and inhibits the activation of macrophages, monocytes, and eosinophils [92]. In experimental models of asthma, a decrease in serum synthesis of IL-4, IL-5, and IL-13 reduces the level in bronchoalveolar lavage fluid, contributing to a reduction in BHR [93].

- Fruits, vegetables, fiber, and their relationship with pulmonary function

In children, a low-fiber diet is associated with chronic inflammation, increased risk of asthma, and respiratory infections, while adequate dietary fiber intake can improve asthma control and support the normal development of lung function. The mechanisms by which dietary fiber influences lung health, especially in the pediatric population, are not completely elucidated [94,95,96]. Higher fruit consumption is significantly correlated with better asthma control, a reduction in the number of emergency room visits, a reduction in absenteeism due to illness, and an increase in quality of life [97]. These benefits are not present with milk or fruit juice consumption, especially if sweetened with sugar. Studies from the last two decades consider the improvement in FEV1 and FVC observed in patients who consume more fruits and vegetables to be a consequence of the effect of antioxidants contained therein [98,99]. Romieu I et al. data showed that a diet with a higher fruit and vegetable index was positively correlated with FEV1 and FVC, and inversely proportional to IL8, i.e., bronchial inflammation [33]. Lower FEV1 and FVC values in children with low fruit juice intake have also been reported by other authors [100].

Dietary fiber indirectly influences lung function in children by acting on the intestinal microbiota and systemic inflammation. Similar to the gut–brain axis, the lung communicates with the gut through the immune system through the gut–lung axis. Usually, consumed dietary fiber is not digested and is fermented by intestinal bacteria, resulting in short-chain fatty acids (butyrate, propionate, acetate) with an important role in modulating the immune response at the pulmonary level through metabolic–immune communication, the gut–lung axis, and reducing inflammation at the pulmonary level [94,95]. Dietary fiber helps the correct development of the immune system in children, balancing the Th1/Th2 response and reducing the risk of allergic respiratory diseases. In adults with asthma, a positive association has been demonstrated between fiber intake and FEV, FVC, and the FEV1/FVC ratio [101]. Studies published in the literature have shown higher FEV1 values on spirometry, thus better lung function, and reduced asthma symptoms in children who consume more whole grains, fruits, legumes, and vegetables [96,102].

### 4.6. Iron 

Altered iron homeostasis is associated with a pro-inflammatory state affecting the immune cell function, promoting the development of allergic disorder like asthma, and influencing the severity through airway inflammation [103]. The direct causality effect is not known, but scientific data support that children suffering from atopic disorders are eight times more likely to have iron deficiency anemia [104,105]. The exact mechanism of asthma pathogenesis is not fully known. Atopy and inflammation are present in asthma and are strongly related to iron metabolism. Inflammation can induce the “functional” iron deficiency with iron being blocked in the deposits and increased consequences on immune cell activity. This type of functional iron deficiency is also present in the context of infection, aggravating the inflammatory response [106].

Other factors like socioeconomic status, nutritional intake, or local airway iron dysregulation may influence both anemia and asthma severity [107], making it difficult to support a causal effect of systemic iron status on asthma risk [103]. 

Recent research shows that individuals with iron deficiency anemia experienced a significantly higher prevalence of asthma [108,109] and also reduced asthma control [110].

- Iron and pulmonary function

In adults, ferritin levels were not associated with FEV1 [108]; in children, decreased serum iron level was related with uncontrolled asthma [111]. Furthermore, low serum iron was associated with airway dysfunction represented as oscillometric respiratory resistance at 5 Hz [112] and hemoglobin or ferritin levels were associated with reduced pulmonary function parameters (FEV1, FVC, FEV1/FVC, PEF) in children with asthma [113], supporting the observation that asthmatic children with anemia have significant lower values of FEV1 in comparison to non-anemic asthmatics [114].

Iron deficiency anemia tends to be more common in children with atopic diseases and asthma. Asthmatic children with iron deficiency anemia have significantly lower values of FEV1 in comparison to non-anemic asthmatics. Low folate levels were associated with asthma in children, but the association with lung function parameters remains debatable.

### 4.7. Dietary Salt

Interventional studies suggest that increased salt intake may increase BHR. A possible mechanism is related to sodium transport flow in smooth muscle cells, which may increase intracellular calcium concentration, impacting smooth muscle tone regulation [115]. The secondary increased release of histamine, through the inhibition of the sodium–calcium exchanger, may play a pro-inflammatory role. The effect of salt on hemodynamics and pulmonary function is also evoked due to the increase in circulating blood volume [116,117]. Salt promotes macrophage chemotaxis, inhibits the suppressive function of Foxp3+ regulatory T cells, and may stimulate TH17 cell differentiation. The influence of salt intake on asthma incidence and possible mechanisms were analyzed in a murine model study [118]. The authors induced allergic inflammation of the airways mediated by aeroallergens and exposed the subjects to a diet with increased sodium intake. They found that it causes changes in serum short-chain fatty acids, stimulates the inflammatory response, with reinforcement of the Th17 phenotype, and induces changes in the intestinal and pulmonary microbiome [118]. The effects were more evident in females.

- Salt intake and pulmonary function

In patients with exercise-induced asthma, a low-salt diet correlates with a smaller increase in eosinophil cation protein, IL-1 and IL-8 in sputum and with better lung function [119]. This beneficial effect was not observed for cysteinyl leukotrienes C4-E4, leukotriene B4, or prostaglandin D2-methoxime [119]. After 2 weeks of increased salt intake, FVC, FEV1, and PEF were lower in patients with exercise-induced asthma [115].

A recent study compared lung function and cardiovascular and renal parameters in 10 young people who exercise with 10 young people who did not exercise (the control group) [120]. Baseline spirometry was better in subjects who exercised. However, after five days of salt loading, FEV1, FVC, and PEF decreased significantly in subjects who exercised, while these parameters remained unchanged in subjects in the control group [120].

### 4.8. Vitamin D

Vitamin D, as part of the micronutrients, has a central role in bone metabolism and normal growth. Recent research has demonstrated the implication of vitamin D in cell differentiation and immunoregulation. Calcitriol, the biologically active form of the vitamin, supports the chemotaxis and the antimicrobial peptide synthesis in monocytes and macrophages. It also induces a state of antigenic tolerance by suppressing the maturation of dendritic cells. It facilitates the activity of regulatory T cells, and it inhibits the differentiation of Th1 and B cells. It also promotes the production of IL-10 and inhibits the IL-2. The active form of vitamin D reduces the transcription of anti-inflammatory Th1 cytokines and contributes to the increased expression of pro-inflammatory Th2 cytokines, IL-4, IL-5, IL-9, and IL-14. The effect on innate immunity is due to the stimulation of cathelicidin expression, an antimicrobial peptide that causes the disintegration of the cell membrane of bacterial organisms, thus reducing the risk of infection. In acquired immunity, vitamin D intervenes in the Th1/Th2 balance, producing anti-inflammatory effects [121]. 

The pathogenic pathways through which vitamin D can influence asthma control and lung function are complex. Its role in calcium metabolism and its immunomodulatory and tract remodeling effects through proliferation or hypertrophy of bronchial smooth muscle cells are recognized [9]. Vitamin D also plays a role in the intestinal microbiome, thereby influencing inflammatory responses to non-pathogenic bacteria in the intestine [122,123,124,125].

Worldwide, more than one billion adults and children have low levels of 25-hydroxyvitamin D (<20 ng/mL) [126]. It is more common in newborns, adolescents, and the elderly, but also in women, especially during pregnancy [2]. The implications of vitamin D deficiency on health are manifold, affecting numerous organs and systems. Current recommendations are to supplement vitamin D in children up to the age of 18 [127].

- Vitamin D and asthma

There is a continuous debate surrounding the potential role for vitamin D in reducing the risk of asthma exacerbation, improving asthma control, and lung function in children and adult patients. 

Many recent studies support the correlation between vitamin D deficiency and asthma [128]. The inverse correlation between serum levels of 25-hydroxyvitamin D3 and asthma control has been widely discussed [9]. At values of <20 ng/mL 25-hydroxyvitamin D, the risk is five times higher, and at values of <30 ng/mL 25-hydroxyvitamin D, the probability was three times higher [129]. 

In a study involving 273 children with asthma, vitamin D deficiency was correlated with the severity of exacerbations and the risk of hospitalization, with no significant relationship with lung function. Vitamin D deficiency was correlated with a lower FEV1/FVC ratio, but no correlation was found with FEV1 [130].

- Vitamin D and pulmonary function

A positive correlation between spirometry parameters (FVC, FEV1, FEV1/FVC) and serum levels of vitamin D was obtained for healthy adolescents, at the age of 15 years. This observation was valid for both pre- and post-bronchodilatation volumes but not for the flow rates at 25, 50, and 75% of exhaled FVC [131]. In asthmatic children, vitamin D was insufficiency associated with lower FEV1 and FEV_1_/FVC values compared with healthy controls [130,132]. Recent studies searched the correlation between vitamin D supplementation and improved lung function in asthmatic patients. In Table 3, we summarize the randomized control trials with two pilot studies that address this topic focusing on pediatric patients.

As shown in recent meta-analyses, the results of these studies are controversial. Chen et al. found in their meta-analysis from 2021 that vitamin D supplementation safely reduced the rate of asthma exacerbation in adults and children but did not improve ACT score or lung function among patients with asthma treated with corticosteroids [142]. In contrast, the meta-analysis performed by Hao et al. suggested that vitamin D supplementation might reduce the FEV1 and FVC in pediatric patients [143]. Furthermore, Fedora et al. found in a recent meta-analysis significant improvement in predicted percentage of FEV1 levels in children with vitamin D supplementation compared to the placebo group [144]. 

These studies exhibit substantial heterogeneity in study design, sample size, population, and outcomes (ACT score, lung function, number of exacerbations, number of hospital admissions, or systemic cortisone treatment). Besides this, several factors can influence nutritional status: genetic, environmental, socioeconomic factors, and recent diet changes or laboratory challenges in evaluating micronutrient status, such as vitamin D.

A positive correlation between spirometry parameters and vitamin D levels was observed in healthy adolescents. In asthmatic children, vitamin D insufficiency was associated with lower pulmonary function compared with healthy controls. This relationship appears to be dose-dependent, with greater lung function deficits observed in children with more severe vitamin D deficiency. The effect of vitamin D supplementation on asthma remains controversial.

### 4.9. Antioxidants (Vitamin A, C, E, Folic Acid, Selenium)

- Vitamin A

Vitamin A is an essential fat-soluble micronutrient that contributes to the formation of pulmonary alveoli [145], in the development of the respiratory tract, with an effect on the integrity of the mucosal surface [146]. Its antioxidant and anti-inflammatory role has been demonstrated in numerous studies. It has anti-proteolytic activity in the bronchial epithelium [147]. Vitamin A deficiency correlates with reduced alveolar septation but also with functional defects [145]. Some authors have demonstrated a correlation with BHR [148,149]. Vitamin A is involved in regulating the expression of numerous genes [150]. The effect of vitamin A supplementation in patients with SCGB1A1 polymorphism on the level of pneumoprotein CC16 has been proven, which has anti-inflammatory effects and is associated with lung function impairment [148,151]. The role of vitamin A in regulating fibroblasts involved in lung growth via the NCOR2 polymorphism pathway is also suggested, with negative associations found in homozygotes for the T allele [148,152,153].

- Vitamin C and vitamin E

A cross-sectional study conducted on 1616 adult residents without respiratory diseases analyzed the relationship between FEV1 and FVC and antioxidant intake. Serum levels of vitamin C, vitamin E, and carotenoids, including β-cryptoxanthin, lutein/zeaxanthin, β-carotene, and lycopene, were analyzed [99]. The authors included questions about smoking as a confounding factor. It was thus demonstrated that both FEV1 and FVC were higher in participants with higher serum levels of these antioxidants [99]. The combined effect of all these antioxidants was also analyzed. Vitamin E, retinol, and b-cryptoxanthin had the closest relationship with FEV1. Vitamin E in this study had an effect independent of vitamin C, although previous data would have suggested an interrelationship between the effects of the two vitamins. This may be related to the fact that vitamin E is predominantly bound to the cell membrane, with a more stable concentration in tissues. On the other hand, vitamin C is soluble, so its transfer into the respiratory epithelial fluid is greater and, at the same time, its serum concentration is lower and less relevant as an indicator of localized tissue antioxidant mechanisms [99].

Vitamin E and zinc intervene in the differentiation of fetal T helper cells, inducing increased secretion of Th1 cytokines to the detriment of Th2, and thus contributing to decreased interleukin (IL)-4 secretion [154,155,156,157].

- Folic acid

Folic acid is responsible for the complex process of DNA methylation, a central epigenetic mechanism regulating gene expression. These epigenetic changes can influence immune cell development, representing the link between folic acid metabolism and asthma pathogenesis [158]. Data regarding folate metabolism and asthma are inconsistent. Some data support the association between asthma prevalence and low serum folate, but dietary folate intake is not consistently linked to asthma risk [159]. Cross-sectional studies including pediatric patients showed that asthmatic patients had lower levels of folate compared with controls [160,161,162,163]. 

Positive significant correlation with lung function parameters FEV1, FVC, and FEV1/FVC was obtained in some studies [160,162,164], but others failed to demonstrate this association [161,163,165]. Altered folate metabolism was also linked to uncontrolled asthma in children, serum folate was significantly lower in severe asthma exacerbation cases compared to mild/moderate cases [160], and associated with increased odds of at least one severe asthma exacerbation, especially in association with vitamin D deficiency [161,162,166]. In a U.S. nationwide study, a high serum ratio between the active and unmetabolized forms of folic acid was associated with decreased odds of asthma, increased FEV_1_ in children and adults, and increased FVC in adults [167].

- Selenium

Nitric oxide and H_2_O_2_ in the exhaled air of asthma patients are markers of oxidative stress that are higher in more severe forms of the disease. Selenium, which is a central component of glutathione peroxidase, the most studied of the 25 human selenoproteins, would have a beneficial effect on lung function and asthma control by protecting cells against oxidative stress. Lower glutathione peroxidase activity and lower glutathione (GSH) concentration reduce oxidative stress in the lungs when selenium intake is increased [168,169,170,171]. The reduction of airway inflammation and mucus production under the action of selenium has been demonstrated in experimental studies [172]. Another possible mechanism by which selenium reduces bronchial inflammation is through its action on naïve CD4+ T cells. One mechanism could be early regulation of gene transcription, knowing that selenium can affect epigenetic events in naïve CD4+ T cells by reducing genomic DNA methylation [169,173]. It has been shown that higher selenium intake shifts the Th1/Th2 balance in favor of Th1/Treg differentiation [169,173]. Thus, pro-inflammatory Th2 cells, namely IL-4, IL-5, and IL-13, which play a central role in allergic asthma, are released in smaller quantities.

The effects of selenium on asthma remain controversial. A recent meta-analysis analyzed selenium levels in four studies involving 3138 patients with asthma and 33,534 non-asthmatic participants [174]. Selenium concentration was lower in asthmatics than in the control group, but the association did not reach the threshold of significance [174].

- Antioxidant nutrients and pulmonary function

A recent study of 10,034 adults without chronic respiratory disease showed that increased intake of vitamins A and K correlates with a significant increase in FEV1 and FVC [145]. An experimental study has shown that vitamin A deficiency causes elastin degradation and increased levels of transforming growth factor beta (TGF-β) in the lung parenchyma [145,150]. A retrospective analysis of lung function in 231 children with cystic fibrosis showed that serum retinol levels correlate positively with FEV1 [147]. Low retinol levels are associated with an increased risk of exacerbation.

A 2017 review suggests that the effect of vitamin C on FEV1 in children may be age-dependent [40], while vitamin E still appears to play a controversial role. Some data show no correlation with lung function, while a randomized trial demonstrated an increase in FEV1 and FEV1/FVC after vitamin E supplementation [40,175]. 

A recent cross-sectional analysis that included 4541 individuals from the US National Health and Nutrition Examination Survey examined the relationship between selenium intake and lung function [169]. Participants included both children and adults, of whom 329 (8%) were asthma patients. A significant positive correlation was found between selenium intake and FEV1, FEV, and the FEV1/FVC ratio in asthma patients [169], with the differences being significantly greater in those with higher selenium intake. Serum selenium concentration correlated with FEV1 and the FEV1/FVC ratio in adults with asthma [176], as did the copper/zinc and copper/selenium ratios. The study also demonstrated a correlation between serum selenium levels and the copper/selenium ratio and the CD4/CD8 lymphocyte ratio [176,177]. Copper may play a role in asthma inflammation both at high concentrations and in circumstances where it is deficient [177]. It is a microelement that acts as a cofactor for enzymes such as cytochrome C oxidase, which is involved in energy metabolism, or Cu-Zn-superoxide dismutase, which is involved in the antioxidant-oxidant balance, and is responsible for the activation of phosphatidylinositol-3-kinase, an enzyme that intervenes in the recruitment of inflammatory cells and stimulates the production and release of IL-6 [178]. Some authors have shown correlations between the level of copper in induced sputum in adults with asthma [179], but without correlating it with FEV1.

The polyphenols and antioxidants contained in fruits and vegetables have anti-inflammatory effects, improving lung function. A diet rich in fiber and low in salt can also improve lung function. Salt ingestion is inversely correlated with lung function parameters, an effect particularly evident in patients with exercise-induced asthma. Selenium intake has been shown to have beneficial effects on lung function, with the copper/selenium ratio being of greater clinical and pathophysiological relevance in some studies. Some authors have reported a beneficial effect of vitamin E supplementation on lung function in children with asthma, but this should be further analyzed in prospective studies before a dietary recommendation can be made.

## 5. Maternal Diet

Primary prevention of asthma and other allergic diseases is a constant concern following the observed upward trend in prevalence. One of the modifiable factors could be nutrition. In utero exposure could influence the development of the respiratory tract and possibly alter the fetal immune response. Maternal nutrition during pregnancy is increasingly recognized as a determinant of immune programming in the offspring [180]. 

Current guidelines acknowledge the biological plausibility of this association but do not provide quantitative recommendations due to limited interventional data [11,180]. EAACI guidelines on the prevention of allergic diseases emphasize the importance of maternal diet during pregnancy but recommend focusing on overall dietary patterns rather than single nutrients [180]. Similarly, the GINA strategy document acknowledges prenatal nutritional factors as potentially modifiable contributors to asthma risk, while noting insufficient evidence to support specific dietary interventions for primary prevention [11]. 

Lung formation and development occur prenatally and early postnatally. There is evidence that alveolarization continues throughout childhood, which would allow for recovery of alveolar growth [148].

### 5.1. Correlation of Maternal Nutrition During Pregnancy with the Onset of Asthma

A meta-analysis of 32 studies (29 prospective cohort studies and 3 retrospective cohort studies) showed that intake of vitamin D, vitamin E, zinc, and dairy is inversely associated with the risk of childhood wheezing. For maternal intake of saturated fatty acids (SFAs) during pregnancy, for both the Mediterranean or Western diet, some studies have shown an inverse association with wheezing, but the data are limited and the evidence is inconsistent. Copper is inversely associated with food allergy, and magnesium and vegetables with eczema. This meta-analysis supports the role of the Mediterranean diet during pregnancy in protecting against asthma and allergic rhinitis [181]. 

From the Avon Longitudinal Study of Parents and Children (ALSPAC) cohort, which included 12,078 pregnant women, 3163 mother–child pairs with information on maternal diet were analyzed [182]. The association with asthma was investigated, as were polymorphisms in maternal antioxidant genes.

- Maternal Mediterranean diet during pregnancy

The beneficial effect of the Mediterranean diet during pregnancy is due to its antioxidant and anti-inflammatory properties of omega-3 PUFA [183,184,185].

The relationship between the Mediterranean diet in pregnant women and the risk of asthma and other allergic diseases in children is reported differently in different studies. Chatzi et al. reports a lower risk of wheezing and atopy associated with the Mediterranean diet in a sample of 460 mother–child pairs from Menorca [186], while another cohort that analyzed 1376 children found no association with wheezing, asthma diagnosis, or atopy [187]. In the largest cohort, which included 8970 children, no relationship was found between adherence to the Mediterranean diet and the prevalence of asthma, eczema, or allergic rhinitis [183]. This diet has an antioxidant effect and may have a protective effect on wheezing and asthma [39].

- Maternal Western diet during pregnancy

In a double-blind, placebo-controlled, parallel-group study that recruited pregnant women from the COPSAC2010 (Copenhagen Prospective Studies on Asthma in Childhood 2010) cohort, the effect of an n − 3 long chain PUFA diet on respiratory health was investigated [184]. A total of 695 children were followed up until the age of 3 and 5 years, both in terms of clinical manifestations and lung function. The authors report significant differences for asthma. The effect was more evident in children whose mothers carried the variant of the gene encoding fatty acid desaturase associated with low levels of eicosapentaenoic acid and docosahexaenoic acid (minor allele G with genotype rs1535) [184].

Saturated fatty acid (SFA) consumption during pregnancy may be correlated with the incidence of wheezing, but intake of MUFA, n3 PUFA, n6 PUFA, or total fat is not significantly associated with asthma [181]. The authors urge us to interpret the results with caution due to the heterogeneity of the studies analyzed.

One of the characteristics of the Western diet is its high salt content. An experimental study has demonstrated the relationship between a mother’s high-salt diet during pregnancy and the complex mechanism by which the lung health of her offspring is affected [43]. The influence on lung transcriptomes has been proven, with significant differences related to sex being described. Thus, in male offspring, increased expression of α-SMA, collagen I, Fn1, and TGF-β was identified, correlating with a higher susceptibility to pulmonary fibrosis and pulmonary candidiasis [43]. In female offspring, a higher risk of pneumothorax was highlighted, correlating with different molecular functions in terms of binding, the catalytic activity of the alpha receptor of platelet-derived growth factor, the activity of the myosin phosphatase regulator, and the molecular translator.

Ultra-processed foods are often packaged in plastic. They contain industrialized chemicals (bisphenols and phthalates) that might contaminate food [41]. Prenatal exposure to bisphenol A, especially during the second trimester of pregnancy, was associated with a high risk of wheezing and asthma in childhood [188]. Prenatal exposure to phthalates might also be linked to childhood asthma [189].

Recent meta-analysis involving 18,326 children from seven cohorts examined the relationship between pro-inflammatory diet and the incidence of wheezing and asthma in children [190]. A moderate correlation between the two was demonstrated.

- Maternal dietary fiber intake during pregnancy

Dietary resveratrol intake during pregnancy is associated with a lower risk of wheezing and allergic rhinitis [191].

Two cohorts studied the mother’s diet and the risk of developing asthma, but although salt is included in Willers, there are no direct results on its role [192,193].

Dietary fiber, a key component of plant-based diets, influences immune responses primarily through modulation of the gut microbiota and its metabolic products, particularly short-chain fatty acids (SCFAs) [194,195]. Proposed mechanisms involve gut microbiota–derived short-chain fatty acids (SCFAs), which modulate fetal immune development through epigenetic and immunoregulatory pathways [194,195]. Dietary fiber is fermented by intestinal microbiota into SCFAs, including acetate, propionate, and butyrate [194,195]. These metabolites can cross the placental barrier and influence fetal immune development through epigenetic mechanisms and modulation of immune cell differentiation. Experimental studies demonstrate that SCFAs promote the expansion of regulatory T cells and suppress Th2-skewed immune responses associated with allergic inflammation [195]. A low-fiber maternal diet may therefore result in reduced SCFA availability, impaired immune tolerance, and increased susceptibility to allergic airway diseases in offspring.

Some observational studies suggest a modest inverse association between maternal fiber intake and allergic rhinitis in offspring, whereas others report no statistically significant relationship [96]. 

Current evidence supports a biologically plausible association between low maternal dietary fiber intake during pregnancy and increased risk of allergic airway diseases, particularly asthma. The consistency between epidemiological findings and mechanistic data from experimental models strengthens this hypothesis [194,195]. Maternal dietary fiber intake during pregnancy may influence the risk of allergic airway diseases in offspring, particularly asthma. Encouraging consumption of fiber-rich foods during pregnancy represents a safe and potentially effective strategy for primary prevention of allergic diseases.

- Maternal vitamin D during pregnancy

The Endocrine Society guidelines recommend empirical vitamin D supplementation for pregnant women [127].

A randomized, double-blind, placebo-controlled study in three centers in the US analyzed the effect of administering 4400 IU of vitamin D versus 400 IU of vitamin D to pregnant women [196]. Mothers who had risk factors (atopy in both the mother and father) for having a child with asthma were selected. In the 806 children followed from birth to age 3, it was found that although children born to mothers with higher vitamin D intake had a 6.1% lower incidence of asthma and wheezing, this was not statistically significant (24.3% versus 30.4%; *p* = 0.051) [196]. They initially had a higher concentration of vitamin D in their umbilical cord blood, but after one year of age, the concentration was similar to that of children born to mothers with low vitamin D intake. The incidence of eczema was similar, but children of mothers with high antenatal vitamin D intake had significantly fewer positive specific IgE results [196]. 

The same group of authors monitored the children until the age of 6 [197]. There were no differences in the incidence of asthma diagnosed at age 6 between children whose mothers had increased antenatal vitamin D intake compared with the control group (20.5% versus 17.1%; 95% confidence interval [CI], −2.5 to 9.5). Of the 360 children with an initial diagnosis of asthma, only 198 had a doctor-confirmed diagnosis at age 6, with the rest classified as recurrent wheezing [197]. The incidence of eczema (26.6% versus 26.3%) and allergic rhinitis (36.3% versus 41.3%) was similar in both groups analyzed. The authors did not analyze postnatal vitamin D intake in these children. Some of those who had wheezing in the first 3 years of life probably had this manifestation correlated exclusively with viral infection, and the difference between its incidence at 3 years compared to older children may be due to the role of vitamin D on antimicrobial peptides [197]. At age 4, impulse oscillometry was performed on 284 children, and at ages 5 and 6, spirometry was performed on 285 children. Total airway resistance and peripheral airway resistance were lower at 4 years of age in children whose mothers had higher vitamin D concentrations at birth, with the difference being significant only for peripheral resistance (95% CI, 0.30 to 0.34). Spirometric parameters, FEV1, and FVC had higher values in those whose mothers had higher vitamin D concentrations, with no statistically significant differences.

However, stratified analysis of data from The Vitamin D Antenatal Asthma Reduction Trial shows that higher vitamin D concentrations in mothers during the first trimester of pregnancy correlate with a lower incidence of asthma [198].

The effect of supplementing with 2400 IU vitamin D versus 400 IU on the incidence of asthma at 3 years was also analyzed in the COPSAC2010 cohort with data available for 521 children [199]. The higher dose of vitamin D was associated with a lower but insignificant incidence of wheezing episodes.

Some studies have suggested that increased intake of folic acid during pregnancy can increase the risk of asthma, possibly mediated by epigenetic mechanisms [173]. Systematic analysis regarding these aspects showed that there is no reason to alter current recommendations for folic acid supplementation during conception or pregnancy based on findings for folate and asthma [166,200,201,202].

- Maternal micronutrients intake during pregnancy

The ALSPAC study demonstrated an inverse association between preformed vitamin A intake and asthma incidence [148]. Of the 8135 children in the ALSPAC cohort, the authors identified 390 (8.6%) cases with asthma. Such data are also suggested by other studies, both in terms of the likelihood of developing asthma and in correlation with its severity [146].

A recent experimental study has shown that antenatal and postnatal selenium deficiency during early breastfeeding causes lung development disorders [203]. The reduction of pulmonary selenoproteins secondary to selenium deficiency in the mother decreases glutathione peroxidase activity, including in the lungs of the newborn, and affects alveolar growth during fetal and neonatal life. This could represent a risk factor for postnatal lung damage [203].

### 5.2. Correlation of Maternal Diet During Pregnancy with Pulmonary Function (Table 4)

- Mediterranean diet versus Western diet

The hypothesis that the Mediterranean diet during pregnancy protects against the risk of developing asthma or other allergic conditions has not been confirmed in the ALSPAC cohort [183]. Lung function was assessed in 8970 children at ages from 7 to 9. The data show that it may have a favorable influence on forced expiratory flow at 25–75% of FVC (FEF25–75%) measured at the age 8.5 years [183]. The analysis also included possible confounders such as maternal age, duration of breastfeeding, smoking, or paracetamol use during pregnancy. The initial analysis and the analysis after excluding confounders showed higher FEV1 and FEF25–75 values in children whose mothers had a higher Mediterranean diet score [183]. Even when assessing lung function at age 15, these differences remain in favor of children exposed to a Mediterranean diet before birth, as they have higher FEF25–75 values [183].

A meta-analysis published in 2022 also evaluated the role of the mother’s pro-inflammatory diet during pregnancy on lung function in the child [190]. There was an inverse correlation between energy-adjusted DII and the FEV1/FVC ratio, but not with FEF25–75. FEV1 and FVC were lower in children born to mothers with a higher DII score.

In a double-blind, placebo-controlled study of pregnant women recruited from the COPSAC2010 cohort, the authors reported no association between long-chain PUFAs intake and lung function [184]. Thus, reduced intake of n − 3 long-chain PUFAs in pregnant women was not correlated with significantly higher FEV1 and FEF25–75% values [184]. Other parameters of respiratory function were also analyzed, such as specific airway resistance and lung-clearance index measured by multiple-breath washout, but the differences were insignificant [184].

A study based on the Viva project cohort analyzed the DII during pregnancy and its relationship with the onset of asthma and lung function [193]. The DII is not a dietary model, as it is calculated based on 28 dietary parameters, which include dietary macro- and micronutrients, as well as bioactive compounds that confer a pro- or anti-inflammatory potential to the diet. In the 1424 children analyzed, a relationship was found with early wheezing and lower FEF25–75 values in children whose mothers had a pro-inflammatory DII [193].

- Maternal micronutrients intake

From the ALSPAC cohort, respiratory function tests were performed on 8915 children aged between 7 and 9 years, and the correlation with maternal consumption during pregnancy of fruit, vegetables, vitamins A, C, and E, zinc, and selenium was analyzed [182]. The premise was that a diet low in antioxidants would contribute to increased inflammation of the airways due to increased susceptibility to oxidative damage. In addition, maternal smoking 3 months prior to pregnancy was also investigated, as it is known to alter lung function in children and is a possible confounding factor. In parallel, DNA samples were analyzed from both mothers’ blood collected during pregnancy and from their children. In children, samples were obtained from umbilical cord blood or venous blood collected at the age of 7 years. Two single nucleotide polymorphisms (SNPs) were identified: an SNP in GSTP1 (G313A, Ile105Val, rs1695) and an SNP in GPX4 (glutathione peroxidase 4; rs713041, at position 718). The only positive association found after excluding confounding factors was between increased maternal zinc intake during pregnancy and higher values of FEV1 and FVC in the child. An interaction between maternal zinc intake and glutathione S-transferase GSTM1 genotype with an effect on FVC was also documented [182]. The intake of fruits, vegetables, or selenium did not reveal any correlations with GST gene polymorphisms and FVC or FEV1 impairment. The consumption of antioxidants, namely fruits, vitamin C, and vitamin E, correlates with higher FEF25–75 values in children, with no statistical differences or evidence of interaction [182]. 

Talaei et al. analyzed the relationship between vitamin A and lung function in children in the ALSPAC cohort. They found a non-linear association with FEV1 and FEF25–75% that does not correlate with maternal vitamin A intake during pregnancy [148].

A longitudinal cohort study conducted on 1861 children whose mothers completed a questionnaire on nutritional intake during pregnancy and who were followed for 5 years [154] also included the measurement of plasma antioxidant concentrations (α-tocopherol) at 12 weeks of pregnancy and at birth. Respiratory function assessment in children evaluated both baseline and post-bronchodilator FEV1 and FVC, PEF, and FeNO. Serum α-tocopherol levels at 12 weeks of pregnancy and low vitamin E intake were not correlated with FEV1 and FeNO, probably due to insufficient lung development at this stage of pregnancy. Both FEV1, baseline and post-bronchodilator PEF, and FeNO at 5 years were weakly correlated with maternal intake of vitamin C, vitamin E, and zinc [154].

The mechanism by which zinc improves FVC is through its pro-antioxidant action on fetal lung growth. The effect was proven in the ALSPAC cohort study and was correlated with the glutathione S-transferase GSTM1 genotype [182]. Experimental studies have shown that fetal lung growth is impaired in rats with zinc deficiency [204]. The effect appears to be mediated by the disintegrin and metalloproteinase 33 gene, which is a zinc-dependent metalloproteinase that increases susceptibility to asthma [154].

**Table 4:** Correlation between maternal nutrient intake during pregnancy and pulmonary function.

	Author, Year of Publication	Pulmonary Function Parameters			
		FEV1	FVC	FEF25–75	PEF
Vitamin A: α or β-carotene	Talaei et al., 2021 [148]	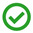 non-linear association		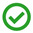 non-linear association	
	Bédard et al., 2018 [182]	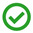 positive correlation	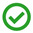 positive correlation	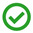 positive correlation	
Vitamin C	Bédard et al., 2018 [182]	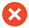 statistically non-significant correlation	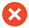	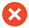 statistically non-significant correlation	
Vitamin D	Litonjua et al., 2020 [197]	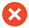 statistically non-significant correlation	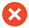 statistically non-significant correlation	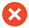	
Vitamin E	Devereux et al., 2006 [154]	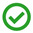 positive correlation	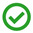 positive correlation		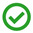 positive correlation
	Bédard et al., 2018 [182]	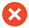 statistically non-significant correlation	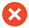	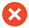 statistically non-significant correlation	
Zinc	Devereux et al., 2006 [154]	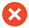	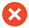		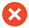
	Bédard et al., 2018 [182]	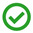 positive correlation	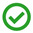 positive correlation	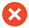	
Selenium	Bédard et al., 2018 [182]	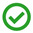 positive correlation	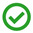 positive correlation	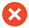	
Vegetable	Bédard et al., 2018 [182]	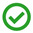 positive correlation	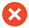	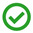 positive correlation	
Fruit	Bédard et al., 2018 [182]	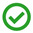 positive correlation	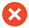	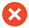	
	Bao et al., 2025 [60]			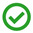 positive correlation	
Dairy	Haas et al., 1991 [80]	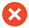			
	Han et al., 2017 [25]	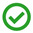 positive correlation	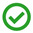 positive correlation		
Fats: MUFAs, PUFAs	Bisgaard et al., 2016 [184]	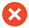		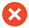	
Mediterranean diet	Bédard et al., 2020 [183]	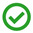 positive correlation		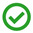 positive correlation	
	Hanson et al., 2020 [193]	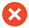	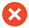	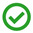 negative association	
DII	Mensink-Bout et al., 2022 [190]		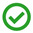 negative association	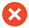	
Western diet	Talaei et al., 2023 [58]	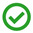 negatively correlated	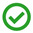 negatively correlated		

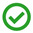
 association; 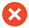 no association; DII, Dietary Inflammatory Index.

## 6. Conclusions and Future Directions 

Unhealthy dietary patterns have been associated with both asthma and obesity. Adherence to a Western diet promotes central obesity and a state of inflammation with a negative impact on pulmonary function in children with asthma. In contrast, the Mediterranean diet, characterized by increased intake of fruits and vegetables, may reduce bronchial inflammation and is associated with better FEV1 and FVC values and better asthma control. 

Dairy products, part of a healthy diet, have a positive effect on pulmonary function.

There is currently substantial evidence supporting better asthma control and improved lung function in patients without vitamin D deficiency compared to those with low serum levels. However, randomized trials are needed to demonstrate the beneficial effect of vitamin D supplementation and to determine the dose required to achieve the maximum benefit on lung function. 

Maternal nutrition during pregnancy could play an important role in the primary prevention of asthma and other allergic diseases. Experimental studies provide evidence that certain micronutrients in the mother’s diet contribute to respiratory tract development and affect the child’s long-term lung function.

One limitation of this review of the literature is the variable study designs of the studies included in the analysis. It is possible that, along with other confounding factors such as socioeconomic status or exposure to secondhand smoke, these factors may affect the accurate interpretation of the data. Similarly, the respiratory function parameters analyzed by different authors vary.

In the future, we propose that greater attention be paid to longitudinal, open-label, controlled studies of modifiable factors, such as nutritional habits and lifestyle, and their potential impact on lung function, asthma control, and patients’ quality of life.

## Figures and Tables

**Table 1:** Studies on the correlation between the Mediterranean diet with pulmonary function tests.

Authors	Year	Type of Study	Study Group	Exposure	Measured Outcomes	Effect Size	95% Confidence Interval	Results
Romieu et al. [33]	2009	Cohort study	Children with asthma aged 6 to 14 years (158 pt) compared with children without asthma (50 pt)	Fruit and vegetable index and a Mediterranean diet index (MDI)	FEV1 FVC FEF25–75 IL8 in nasal lavage Airway inflammation-FeNO and exhaled breath pH	0.058 (0.029) vs. −0.016 (0.047) 0.075 (0.032) vs. −0.025 (0.052)		Children in the highest intake category of the MDI index had a 15.3% higher FEV1 and a 16.5% higher FVC than children with the lowest cate- gory
Li J et al. [38]	2024	Cross-sectional	Adults over 20 years, 1316 with asthma, 8062 without asthma,	Omega-3 polyunsaturated fatty acid consumption	FEV1 FVC FEV1/FVC	β = 10.65 FEV1 β = 22.52	–15·91, 37·22 –5·59, 50·62	Higher lung parameters in those with higher omega-3 PUFA intake
Rice JL et al. [37]	2015	Case–control study	Children with asthma aged 9 to 19 years (287 pt) compared with children without asthma (96 pt)	Mediterranean diet score (MDS) Food frequency questionnaire and	FEV1 FVC FEV1/FVC Asthma Control Test (ACT)	β = 0.18	−3.2, 3.6	No association between MDS scores and asthma control, FEV1
Castro-Rodriguez et al. [39]	2017	Case–control study	1784 children (4.1 ± 0.8 years)	Mediterranean diet	Current wheeze	adjOR = 0.54	0.33–0.88	Lower incidence with Mediterranean diet

**Table 2:** Studies correlating the Western diet with pulmonary function tests.

Authors	Year	Type of Study	Study Group	Number of Children	Exposure	Measured Outcomes	Effect Size	95% Confidence Interval	Results
Han et al. [25]	2017 Puerto Rico	Case–control study	Children with asthma (mean age ± SD = 10 ± 2.6 years) compared with children without asthma (mean age ± SD = 10.5 ± 2.7 years)	678	High saturated fats/processed foods	FEV1 FVC FEV1/FVC ≥1 severe asthma exacerbation	OR ≈ 1.3–1.6	~1.05–2.2	High risk of asthma exacerbations
Talaei et al. [58]	2023 United Kingdom	Longitudinal	Children at 7 years, with dietary patterns followed since birth	2950	Processed foods	FEV1 FVC FEF25–75	OR ≈ 1.2–1.4	~1.01–1.6	High risk of asthma incidence
Rodriguez et al. [59]	2024 Portugal	Cross-sectional	Children aged 7 to 12 years	660	High adherence to protective healthy diet	Airway inflammation- FeNO Airway reversibility and lung function-before and 15 min after inhalation of 400 μg of Salbutamol-FEV1	OR ≈ 0.70–0.85 β ≈ −10% to −20% FeNO	~0.55–0.95 ~−5% to −25%	Protective effect of healthy planetary diet
Bao et al. [60]	2025 China	Observational Cross-sectional	Children with average age 11 years	6276	Western dietary pattern	FEV1/FVC	OR ≈ 1.2–1.5	~1.05–1.7	High risk of airflow limitation

**Table 3:** Vitamin D supplementation and lung function in asthmatic patients.

Author	Year/ Location	Population	Number	Intervention	Outcome	Observations
Majak et al. [133]	2011 Poland	Children with asthma	48	Vitamin D (500 IU/day), 6 months versus placebo	The improvement in FEV_1_ was the same for both groups	Patient and controls received inhaled budesonide
Bar Yoseph et al. [134]	2014 Israel	Children with mild asthma and insufficient vitamin D (<30 ng/mL)	39	Vitamin D 14,000 IU once weekly, 6 weeks versus placebo	There was no change in FeNO levels or FVC, FEV1, FEF25–75%, and FEV1/FVC following treatment	No chronic treatment
Saba Arshi et al. [135]	2014 Iran	Mixet group 10–50 years with asthma	130	Vitamin D (100,000-U bolus intramuscularly plus 50,000 U orally weekly) 8–24 weeks versus placebo	- FEV_1_ improved significantly in both groups after 8 weeks, without significant difference - FEV_1_ was significantly better in the intervention group than controls after 24 weeks	Chronic treatment for both groups’ budesonide or budesonide plus formoterol
Tachimoto et al. [136]	2016 Japan	Children with asthma	89	Vitamin D (800 IU/day) 2–6 months versus placebo	- The proportion of patients with a PEFR < 80% predicted was significantly less in the vitamin D group	94% used long-term inhaled corticosteroids or LTRA before the trial began vitamin D levels were around 30 ng/mL in most of cases
Kerley et al. [137]	2016 Ireland pilot	Children with asthma	44	Vitamin D (2000 IU/day) 15 weeks versus placebo	No significant, improvement in lung function, FEV1	
Han et al. [138]	2021 USA	Children with asthma and insufficient vitamin D (<30 ng/mL)	176	Vitamin D 4000 IU 48 weeks versus placebo	No significant, changes in lung function, FEV1	Chronic treatment with inhaled fluticasone propionate
Lewis et al. [139]	2021 USA pilot	Children with asthma	30	Vitamin D 1000 IU/day 12 months versus placebo	No significant, changes in lung function, FEV1	
Thakur et al. [140]	2021 India	Children with moderate persistent asthma	60	Vitamin D 2000 IU/day 12 weeks versus placebo	No significant, changes in lung function, FEV1	
Napatsayod Swangtrakul et al. [141]	2022 Thailand	Children with well-controlled asthma	84	Vitamin D2 300,000–600,000 IU (based on weight) divided in 5 days in the first visit then a maintenance dose of 20,000 IU every 2 weeks started at 1-month visit versus placebo	No significant differences of forced oscillation parameters among groups No correlation of serum vitamin D with % predicted of forced oscillation technique measures	

## Data Availability

Not applicable.

## Abbreviations

The following abbreviations are used in this manuscript:

ALSPAC	Avon Longitudinal Study of Parents and Children
ACT	asthma control test
BHR	bronchial hyperresponsiveness
COPSAC	Copenhagen Prospective Studies on Asthma in Childhood 2010
DII	Dietary Inflammatory Index
FEF25–75	Forced expiratory flow between 25 and 75% of FVC
FEF50	Forced Expiratory Flow at 50% of FVC
FeNO	fractional exhaled nitric oxide
FEV1	forced expiratory volume in one second
FVC	forced vital capacity
GPx	glutathione peroxidase
GST	glutathione S-transferase
GSH	glutathione
IL	Interleukin
LTRA	Leukotriene Receptor Antagonist
MEF25	Mean Expiratory Flow rate at 25% of vital capacity
MEF50	Mean Expiratory Flow rate at 50% of vital capacity
MMEF	maximal mid expiratory flow
MBW	multiple breath washout,
MUFAs	Fats monounsaturated fatty acids
PEF	peak expiratory flow
PUFAs	n3 or n6 polyunsaturated fatty acids
SCFA	short-chain fatty acids
SFAs	saturated fatty acids
α-SMA	Alpha-Smooth Muscle Actin
SNP	single nucleotide polymorphisms
TGF-β	transforming growth factor beta
TNF	tumor necrosis factor
TSLP	thymic stromal lymphopoietin
